# How acceptable do parents experiencing mental health challenges find e-Health interventions for mental health in the postnatal period: a systematic review

**DOI:** 10.1186/s12884-022-05070-7

**Published:** 2022-10-12

**Authors:** Rosie Attard, Jane Iles, Rose-Marie Satherley

**Affiliations:** School of Psychology, Department of Psychological Interventions, University of Surrey, GU2 7XH Guildford, England, UK

**Keywords:** postnatal mental health, remote interventions, e-Health, review

## Abstract

Poor mental health in the postnatal period is experienced by high numbers of parents, with a high associated cost to society, however accessing therapeutic support during this time is complicated by parenting commitments. This has been further compounded by the covid-19 pandemic, where access to traditional therapy has been impacted. A lack of access to support for poor mental health in this period can have long term impacts on both the parents and their child. E-Health provides a potential solution to parents accessing support during this period by providing a convenient and flexible intervention which overcomes the barriers of traditional face-to-face therapy. However, without investigating the acceptability of such support for parents, it is not possible to predict uptake and consequent effectiveness. The current review synthesizes data available on acceptability of e-Health interventions in the post-natal period, finding that parents valued e-Health interventions however considerations must be made to certain, key areas impacting the acceptability of these interventions for parents. An element of therapist support and individualised content was preferred, along with a smooth user experience. Parents valued that e-Health fit into their routines and provided anonymity in their interactions. Further research needs to be completed into acceptability for minority social and ethnic groups where access and preference may differ.

**Supplementary Information:**

The online version contains supplementary material available at 10.1186/s12884-022-05070-7.

## Introduction

The first postnatal year is a time of change which can increase the risk of poor parental mental health [1]. Estimates indicate 10–15% of mothers in high-income countries and 10–41% of mothers in lower- and middle-income countries are typically affected by poor mental health, and this figure is thought to have risen to 20–64% of mothers giving birth during the COVID-19 pandemic [2, 3]. Whilst research in this area has traditionally focused on maternal mental health, specifically postnatal depression, there is evidence for a range of mental health difficulties, regardless of gender [4, 5]. According to Schumacher, Zubaran & White’s literature review, up to 25% of fathers experience postnatal depression, and this increases to 24–50% of fathers when their partner also experiences postnatal depression [6].

The impact of poor mental health in the postnatal period affects both the family and the wider health system. At the family level, poor parental mental health in the postnatal period influences the child’s social-emotional development, the mother-baby bond, as well as the mother’s satisfaction with their spousal relationship [7, 8]. At the health system level, perinatal mental health has considerable financial implications. In the UK, Bauer, Knapp & Parsonage found that the long-term cost of one case of perinatal depression is approximately £74,000, with 28% of the costs relating to the mother and 72% relating to the child [9]. Despite the negative consequences of poor perinatal mental health, most parents do not receive psychological support for their mental health [10].

To address increasing mental health need, and facilitated by the COVID-19 pandemic, the development and provision of mental health support delivered remotely via the internet, also referred to as ‘e-Health’, has increased [11]. E-Health includes psychological therapies delivered via remote means such as telephone or videoconferencing, internet-based interventions, smartphone apps, wearables, and virtual reality [11]. Many countries are now recommending digital health care as a first step to addressing perinatal mental health, and this has been embedded within both policy and practice [12, 13].

e-Health solutions present several opportunities for the provision of perinatal mental health support. Firstly, e-Health solutions may be one way to increase the reach of psychological services, delivering support to those who wouldn’t traditionally be able access face-to-face services due to geographical restrictions [14]. Given e-health holds no geographical boundaries, it may facilitate flexibility in the time and location of service delivery [15]. This is important for postnatal parents, who may be less mobile and have less free time available to them due to commitments to their newborn child. Secondly, e-health apps can record detailed data on patient activity, including sleep patterns, and self-rated mood scores. Additionally, e-health may provide a more cost-effective solution to postnatal mental health, whilst promoting patients’ involvement in their care.

Despite technological advancements and the anticipated benefits of e-health for parents in the postnatal period, these solutions are often designed without patient or clinician involvement. A systematic review exploring the efficacy of e-Health interventions for parent’s postnatal mental health, found positive effects on mental health outcomes across the included interventions [16]. However, this review did not include information on parental acceptability of e-Health interventions. Van Den Heuvel et al.’s review further demonstrated the utility of e-Health interventions in the perinatal period whilst emphasizing the need to consider parental acceptability [17]. Therefore to inform the development of further postnatal e-health interventions for mental health, this review provides a detailed synthesis of patient acceptability of e-health interventions.

Aim: This systematic review aims.

1. To synthesise the existing qualitative data on acceptability of these e-Health support systems for new parents.

## Method

### Registration

The systematic review was registered on PROSPERO (reference: CRD42021253507).

### Search strategy and selection criteria

Articles were obtained from four databases in May 2021: APA PsycInfo, MEDLINE, APA PsycArticles, Psychology and Behavioral Sciences Collection. Reference lists of relevant papers were also reviewed. Search criteria consisted of word related to four key areas; ‘postnatal’, ‘intervention’, ‘mental health’ and ‘internet based’. Additional search terms related to ‘qualitative approaches’ were included, following the search criteria used by [18]. A list of the full search terms can be found in Appendix 1. Qualitative studies that met the criteria below, were eligible for inclusion; there were no date restrictions.

- **Study design**: Qualitative; interviews, focus groups and qualitative data captured by any other means.
- **Population**: parents who had a form of direct contact with an e-Health intervention for postpartum mental health difficulties (typically the postnatal period is defined as one year following childbirth however here the description of postnatal time frame is defined by individual studies).
- **Intervention**: psychological interventions for use by parents in the postnatal period administered via e-Health means.
- **Outcome**: The population’s qualitative experiences of intervention acceptability.

### Study selection and data extraction

**Fig. 1:**
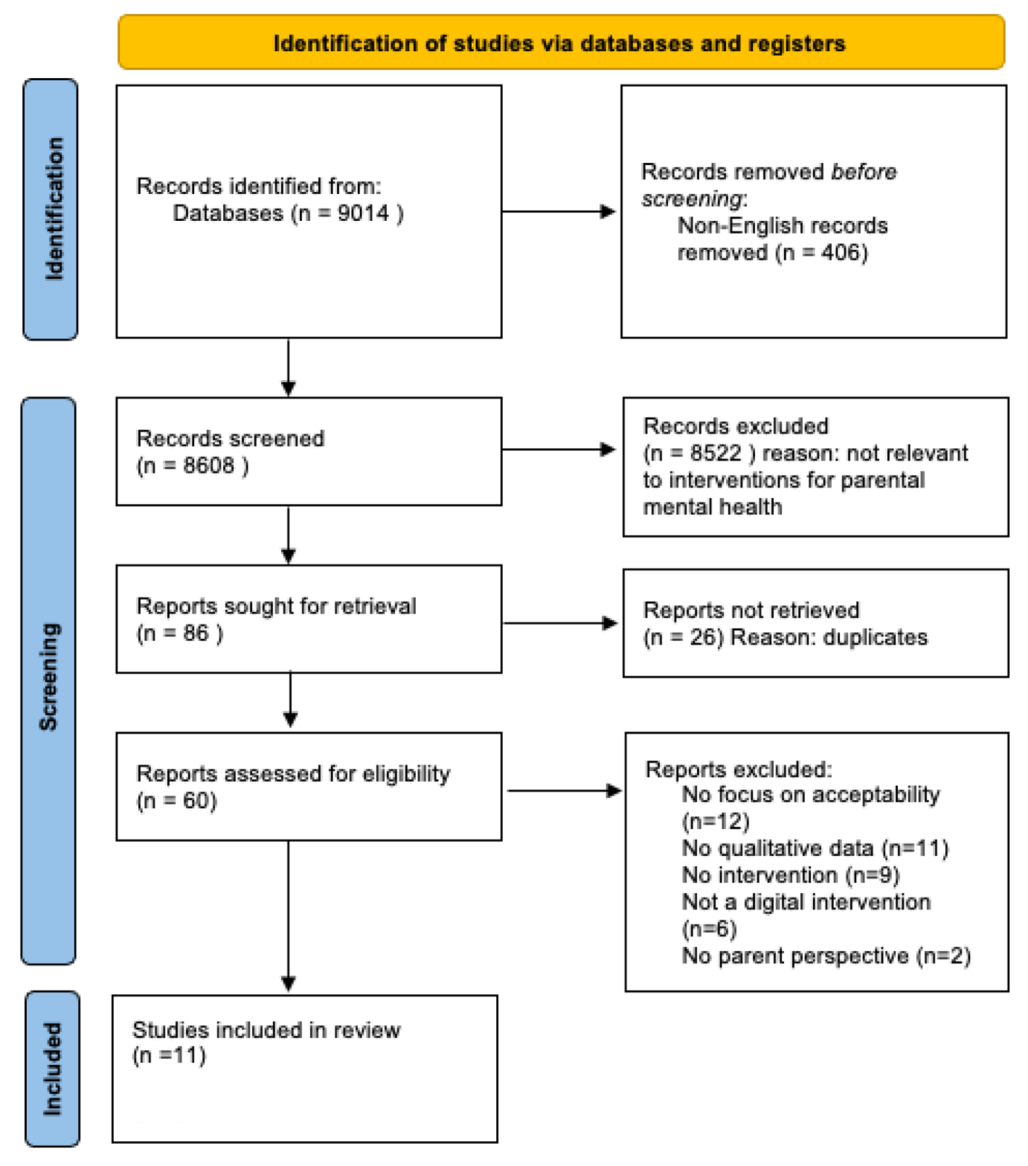
Study selection process

Titles and abstracts were screened for eligibility and full text versions of relevant papers were reviewed by the first author who extracted information relating to study design, population, intervention characteristics (language, problem area, intervention format, structure of the program, therapeutic approach) and outcomes. A second reviewer screened full text articles to reduce the likelihood of bias in the selection process. Where any discrepancies in opinion occurred, a third researcher was consulted for their opinion. See Fig.1 for a PRISMA diagram reporting this process [19].

### Quality assessment

The Critical Appraisal Skills Programme qualitative research quality checklist (see Appendix 2) was used to assess the quality of each study [20]. The CASP [20] quality checklist does not impose a quality cut-off or rating but instead encourages the reviewer to consider numerous salient elements of the methodologies and reporting quality. Therefore, in this review no arbitrary cut-off for quality was imposed, rather the papers were considered for their overall quality.

### Data synthesis

Descriptive summaries of the design, characteristics, and results of included studies were completed. Primary data, and the narrative commentaries on these data were extracted verbatim and read numerous times to allow the comparison of data within and across articles. Analysis of the primary data was completed using thematic synthesis, examining the data line-by-line to code and identify key themes across studies [21]. The key themes were then summarized in a table and scrutinized across studies. This process was conducted by the first author and regularly discussed with the research team throughout.

## Results

The search resulted in 9,014 papers, of which 11 were eligible for inclusion. Due to being written in a language other than English, 406 papers were excluded pre-screening. Following screening, 8522 papers were excluded due to not being relevant to interventions to parental mental health. Full papers were retrieved and screened for 86 papers, and 26 duplicates were removed. Of the remaining 60 papers 49 were excluded due to not meeting study criteria. An overview of the study selection process can be found in Fig.1, and the included papers in Table1.

**Table 1:** Data extraction

Author and Country	Population	Intervention characteristics	Data collection & analysis:	Summary of Themes	Additional Features of Interest	Quality Scoring
1. Ashford, M. T., Olander, E. K., Rowe, H., Fisher, J. R., & Ayers, S. (2018). [Australia]	**N**: 89 (Treatment = 46, waitlist = 43) **Recruitment method**: via social media **Participants**: Women in the postpartum period (< 12 months since childbirth) with mild to severe anxiety.	**Name: What Am I Worried About (iWaWa)** **Language**: English **Intervention format**: 9 online modules with optional weekly telephone support from a therapist **Intervention target**: mild to severe anxiety **Therapeutic approaches**: Cognitive behavioural therapy and mindfulness	**Data collection**: Semi structured telephone interviews post intervention **Analysis**: Thematic analysis	• **Presentation and format** • Subthemes: Accessibility, anonymity, support option, website usability, support format. • **Content** Subthemes: Learning CBT & mindfulness, relevance and helpfulness, appropriateness, format issues • **Helpfulness**	High dropout (only 2 women completed all 9 modules). Deemed not feasible in its current format	
2. Baker-Ericzén, M. J., Connelly, C. D., Hazen, A. L., Dueñas, C., Landsverk, J. A., & Horwitz, S. M. (2012). [Mexico]	**N**: 79 **Recruitment method**: Recruited from prior research list through obgyn clinics. **Participants**: Low-income Latina women receiving perinatal services in publicly funded OB/GYN community clinics located in Southern California, who screened positive for major depression.	**Name: Perinatal Mental Health Model (PMH)** **Language**: Spanish / English **Intervention format**: Maternal Health Advisor delivers intervention via telephone, regular contact with the advisor throughout intervention. Intervention content included 6 modules addressing psychoeducation around normalizing depression, emotional support, CBT strategies, coping and stress management, referrals. Duration of the intervention was tailored to women’s needs. **Intervention target**: depression **Therapeutic approaches**: Psychoeducation, CBT	**Data collection**: Recording of comments by mothers during the telephone intervention **Analysis**: No description of analysis provided	• Positive comments about understanding of depression • Positive statements about PMH program • Appreciation for phone conversations • Specific techniques that were helpful to them		
3. Barrera, A. Z., Aguilera, A., Inlow, N., & Servin, J. (2020). A preliminary study on the acceptability of a brief SMS program for perinatal women.Health informatics journal,26(2), 1079–1087.	**Country**: metropolitan, urban area of the United States **N**:10 (5 pregnant, 5 postnatal, 7 provided qualitative feedback) **Recruitment method**: Flyer posting at general public bulletin boards (e.g. grocery stores) and at community agencies and websites or blogs (e.g. Facebook, clinics) serving perinatal women **Inclusion**: women who were English- or Spanish-speaking, pregnant or up to 1 year postpartum, and who were willing to receive mood management text messages were eligible to participate	**Name: BabyText Program** **Language**: Spanish and English **Intervention format**: SMS messaging. 31 SMS messages delivered to each woman. BabyText intervention tips were sent every 2 days following enrollment, over the course of 69 days. **Intervention target**: Mood disorders, primarily anxiety **Therapeutic approaches**: CBT **Therapist contact**: No additional contact **Duration of intervention**: 69 days	**Data collection**: Online questionnaire with open-ended questions post intervention. **Analysis**: Grounded theory analysis used	• **Helpfulness of intervention tips** Positive, helpful, improved depressed mood. Simplistic and common sense Need to personalize tips • **Application of intervention content** Majority reported being able to apply and utilise the tips One said vague tips difficult to put into practice Personalisation with application mentioned again.		
4. Danaher, B. G., Milgrom, J., Seeley, J. R., Stuart, S., Schembri, C., Tyler, M. S., … & Lewinsohn, P. (2013). MomMoodBooster web-based intervention for postpartum depression: feasibility trial results.Journal of medical Internet research,15(11), e242.	**Country**: USA & Australia **N**: 53 **Recruitment method**: identified via birth records, nurse/health professional referrals, online advertisements, and news stories to local university and hospital settings. **Inclusion**: <9 months postpartum, ≥ 18 years of age, home Internet access and use of personal email, and an EPDS score from 12–20 or a Personal Health Questionnaire (PHQ-9) score from 10–19. These ranges were chosen to identify women with mild to moderately severe depression.	**Name**: Mom Mood Booster **Language**: English **Intervention target**: postpartum depression **Intervention format**: interactive, guided web based intervention. 6 sessions available weekly: Module content: (1) Getting Started, (2) Managing Mood, (3) Increasing Pleasant Activities, (4) Managing Negative Thoughts, (5) Increasing Positive Thoughts, and (6) Planning for the Future. **Therapeutic approaches**: This treatment model combines cognitive and behavioral strategies to address pessimism, attributions for failure, low self-esteem, low engagement in pleasant activities, social withdrawal, anxiety, and low social support. **Therapist contact**: Coaching contact after each weekly session. **Duration of intervention**: 6–12 weeks with 6–12 coach calls	**Data collection**: Program helpfulness and usability assessed 3 months post intervention through online questionnaire. **Analysis**: No clear analysis described, raw data presented.	• Raw data presented in response to questions about helpfulness • Gave private time to complete intervention • Focus on self • More equipped to manage mood and emotions • Phone calls keep you on track • Coach calls helped to have reminder to log in, reaffirmed things in the course, ‘personal’ feeling, caring, tie the whole programme together, makes you accountable.		
5. Pugh, N. E., Hadjistavropoulos, H. D., & Fuchs, C. M. (2014). Internet therapy for postpartum depression: a case illustration of emailed therapeutic assistance.Archives of women’s mental health,17(4), 327–337.	**Country**: Canada **N**: 1 **Recruitment method**: Convenience sample **Inclusion**: N/A	**Name**: N/A **Language**: English **Intervetion target**: postpartum depression **Intervention format**: email therapy **Therapeutic approaches**: CBT **Therapist contact**: throughout via email	**Data collection**: Feedback sought via email in open ended questions **Analysis**: No analysis, raw data presented.	“I very much enjoyed working online as I could read and do activity planning, relaxation etc. on my own and with my own schedule. I appreciated the weekly emails from Nicky (online therapist) and that she put in lots of time to help me address and identify my causes for anxiety and depression”		
6. Pugh, N. E., Hadjistavropoulos, H. D., Hampton, A. J., Bowen, A., & Williams, J. (2015). Client experiences of guided internet cognitive behavior therapy for postpartum depression: a qualitative study.Archives of women’s mental health,18(2), 209–219. Chicago	**Country**: Canada **N**: 24 **Recruitment method**: newspaper articles and newsletter editorials, radio and television appearances, informal presentations at support groups, information booths, posters at physician offices, and notices posted on relevant websites (e.g., Facebook and community websites). **Inclusion**: (a) a score of 10 or higher on the Edinburgh Postnatal Depression Scale (EPDS; Cox et al. 1987), (b) a child under 1 year of age, (c) access to a computer with the internet, (d) willingness to have a family physician or medical clinic notified of their participation in the program, (e) over the age of 18 years, and (f) residing in the same province as the internet therapy program.	**Name**: TAICBT **Language**: English **Intervention target**: postpartum depression **Intervention format**: Therapist assisted internet CBT Each of the 7 modules included a mood rating, an open-ended check-in submitted to the internet therapist, and psychoeducation focused on the module’s topic. The modules presented text, graphics, animation, audio and video **Module content**: (a) psychoeducation on maternal depression, (b) activity planning, (c) relaxation, (d) thinking styles, (e) cognitive restructuring, (f) problem solving, and (g) relapse prevention. **Therapeutic approaches**: CBT **Therapist contact**: clients received one e-mail a week from their assigned internet therapist.	**Data collection**: Online questionnaire **Analysis**: thematic analysis. 2x researchers/coders and a 3rd analyst	• **Positive**: Experience with int. therapist Value of iCBT content General value of internet therapy • **Challenges**: Logging on Fast pace Mother Role Drawbacks of no f2f • **Future Direction**: Connecting with mums through internet forum		
7. Shorey, S., & Ng, E. D. (2019). Evaluation of a technology-based peer-support intervention program for preventing postnatal depression (part 2): qualitative study.Journal of medical internet research,21(8), e12915.	**Country**: Singapore **N**: 20 (10 control, 10 intervention) **Recruitment method**: **Inclusion**:	**Name**: Peer Support Intervention Programme (PIP) **Language**: English **Intervention target**: postnatal depression **Intervention format**: weekly telephone calls with peer volunteers **Therapeutic approaches**: peer support **Duration of intervention**: 4 weeks	**Data collection**: Qualitative semi structured interviews with 20 mothers (10 control, 10 intervention) **Analysis**: Analysed using thematic analysis by 2 researchers	• Postnatal experience Bouncy ride Way forward • Evaluation of PIP Valuable Flexible Supportive Building blocks of a good relationship Lessons learned and road ahead	Also evaluated from peer supporters perspective but this will not be included in the review	
8. O’Mahen, H. A., Grieve, H., Jones, J., McGinley, J., Woodford, J., & Wilkinson, E. L. (2015). Women’s experiences of factors affecting treatment engagement and adherence in internet delivered Behavioural Activation for Postnatal Depression.Internet Interventions,2(1), 84–90.	**Country**: UK **N**: 17 **Recruitment method**: Recruited online for a larger study via advertisements on website banners, ‘new “Helping with Depression” treatment study’, on the UK Netmums parenting website front page and on their PND chat room page (www.netmums.co.uk), through the Netmums’ newsletter, via emails to women who had posted on the PND chat room in the previous 12 months, and via the Netmums Twitter and Facebook sites.	**Name**: NetMums ‘Helping with Depression’ **Language**: English **Intervention target**: postnatal depression **Intervention format**: 12 session behavioural activation and relapse prevention open access self-help internet intervention. The content included interactive exercises paired with extensive worked examples. Alongside NetMums ‘meet a mum’ feature, which allowed women to connect with other women in their local area. **Therapeutic approaches**: BA functional analytical framework (Addis & Martell,2004). **Therapist contact**: Weekly phone call support from mental health workers with undergraduate degrees and 1 year of further clinical qualification in psychological therapies under the UK Improving Access to Psychological Therapies (IAPT) training scheme.	**Data collection**: Semi structured interviews over the telephone, approx. 60min each. **Data analysis**: Content analysis using 3 analysts. NVivo 9 software	• Relevance to life • Unrealistic expectations of motherhood • Double stigma • Barrier – hopeless mentality • Negative experience with previous treatment • Barrier – inadequate support network • Suggestions of treatment improvement: interaction, individualized, therapeutic support		
9. Seshu, U., Khan, H. A., Bhardwaj, M., Sangeetha, C., Aarthi, G., John, S., … & Raghavan, V. (2020). A qualitative study on the use of mobile-based intervention for perinatal depression among perinatal mothers in rural Bihar, India.International Journal of Social Psychiatry, 0020764020966003.	**Country**: India **N**: 12 **Recruitment method**: Twelve women who were screened to be positive for postnatal depression and received the mobile phone-based intervention, IVRS, in the larger research project were approached for the interview. Nine of the twelve consented to participate.	**Name**: Interactive Voice Response System (IVRS) **Intervention target**: perinatal depression **Intervention format**: Mobile phone audio recordings - seven episodes (audio dramas) fortnightly. Women would receive a call where they would hear the short plays. Module content: The content was based on seven themes namely, Nutritional intake, ante-natal care practices, well-being through exercises and meditation, gender issues (family planning and domestic violence) and stress management. **Therapeutic approaches**: details in other study paper not yet published.	**Data collection**: 12 in depth interviews and 1 focus group **Data analysis**: Thematic analysis of transcripts, using inductive and deductive procedure outlined by Braun and Clarke (2013)	• Acceptability • Userbility • Community Participation • Cost • Preference to either intervention		
10. Avalos, L. A., Aghaee, S., Kurtovich, E., Quesenberry Jr, C., Nkemere, L., McGinnis, M. K., & Kubo, A. (2020). A Mobile Health Mindfulness Intervention for Women With Moderate to Moderately Severe Postpartum Depressive Symptoms: Feasibility Study.JMIR Mental Health,7(11), e17405.	**Country**: California, USA **N**: 27 **Recruitment method**: Clinician referral - Women seeking postpartum care were recruited from 7 of the 44 KPNC obstetrics and gynecology clinics. **Inclusion**: Women aged at least 18 years, within 6 months of giving birth, with a PHQ-9 score of 10 to 19 (indicating moderate to moderately severe depressive symptoms), English-speaking, with access to a smartphone, tablet, or computer with internet access were eligible for the study. Women who engaged in regular mindfulness, meditation, or yoga practice 3 or more times per week or enrolled in a mindfulness program were excluded.	**Name**: Headspace **Language**: English **Intervention target**: postpartum depression **Intervention format**: Self-paced mobile app; the women were asked to use the app for 10 to 20min a day during the 6-week study period. **Therapeutic approaches**: Mindfulness **Therapist contact**: When the study staff noted that a participant had completed fewer than 3 sessions in the past week, they called the participant to remind her to use the app. **Duration of intervention**: 6-weeks	**Data collection**: semi structured interview. **Analysis**: Inductive thematic analysis by 2 primary coders. A third coder reviewed transcripts to ensure accuracy. NVivo 9 software used.	• All participants planned to continue their mindfulness practice post intervention. • Women felt that it was easy to use, ariety of options available, convenience of app based intervention. • Some participants would like different voice options. • Some preference to start at different levels depending on previous experience • Request for more check ins • Commonly mentioned benefits were: improved stress management, reduced anxiety, improved sleep, and increased physical activity.		
11. Hensel, J. M., Yang, R., Vigod, S. N., & Desveaux, L. (2020) Videoconferencing at home for psychotherapy in the postpartum period: Identifying drivers of successful engagement and important therapeutic conditions for meaningful use.Counselling and Psychotherapy Research.	**Country**: Ontario, Canada **N**: 12 **Recruitment method**: convenience sample via postnatal clinics during covid-19 **Inclusion**: a)symptoms of depression and or anxiety and were referred to initiate psychotherapy during the recruitment period or were already receiving a psychotherapy with a minimum of 3 months of planned treatment remaining; b) were at least 18 years of age; c) had access to a video enabled device and a high speed internet connection in their home; d) had an active email address	**Name**: N/A **Language**: English **Intervention target**: mood/ anxiety disorders **Intervention format**: remote therapy via videoconference **Therapeutic approaches**: cognitive behavioural therapy and interpersonal therapy approaches **Therapist contact**: for every session, as frequently as once per week depending on the patient need. **Duration of intervention**: 3 months	**Data collection**: semi structured telephone interviews using interview schedule (not provided) **Data analysis**: Thematic analysis using an inductive, constant comparative approach. Reviewed and coded by 2 members of the research team	• **Themes**: 1) initial willingness to engage with VC 2) technological compatibility 3) good patient fit • **Therapeutic considerations**: 1) An initial in person meeting when possible 2) Matching therapy format to the clinical situation 3) Attention to home environment 4) Clear therapy frame		

### Study characteristics

Most studies were completed in the United States of America (4/11, 36%), followed by Canada (3/11, 27%), Australia (2/11, 18%), and the United Kingdom (1/11, 9%), and India (1/11, 9%). Sample sizes ranged between 1 and 79 parents (100% female). Qualitative data collection methods included semi-structured interviews (5/11, 45%), focus groups (1/11, 9%) online questionnaires/email (4/11, 36%) and spontaneous comment recording (1/11, 9%). Analytical approaches included thematic analysis (6/11, 54%) aiming to identify themes across data, content analysis (1/11, 9%) which considers the presence of words and concepts within the data, and grounded theory (1/11, 9%) which focuses on constructing hypotheses and theory throughout the data. Three studies presented the raw qualitative data as part of a larger study involving quantitative methodology [22–24].

### Intervention characteristics

Characteristics of e-Health interventions varied substantially across studies. Most interventions (8/11, 72%) were designed to target postnatal depression, with the remaining three (27%) primarily focusing on anxiety [1, 3, 11]. Interventions comprised of online modules (5/11, 45%), telephone (2/11, 18%) or video (1/11, 9%) intervention with a therapist, SMS support (1/11, 9%), email intervention with a therapist (1/11, 9%) and audio recordings (1/11, 9%). Interventions also varied in the presence or intensity of therapist or coach contact with participants. In three studies participants had no contact with therapists throughout their intervention [25–27], most commonly, participants received weekly contact with a coach or therapist (6/11, 54%). The professional background of the coaches and therapists ranged from researchers trained in the intervention [23, 28] doctoral trainee psychologists [24, 29] mental health advisors [22], peer volunteers [30] and unspecified therapists [31, 32].

Therapeutic approaches differed between studies. 6/11 (54%) authors reported using Cognitive Behavioural Therapy (CBT). The remaining studies used a range of mindfulness, psychoeducation [22, 31], peer support [30], behavioural activation [29] and interpersonal psychotherapy (IPT), [22]. Full details can be found in Table1. One study [31] which circulated audio recordings like a soap drama, did not specify an approach. The intention in this study was to normalize mental health difficulties, and to support women in an area where postnatal depression is heavily stigmatized. For the purposes of this review, this approach has been classified as psychoeducation.

### Quality assessment

Overall, the quality of the papers was considered acceptable to high; all papers included a clear description of the aims and methods of the research and a clear statement of findings with justification of the chosen methodology and recruitment strategy. However, all but 1 of the papers [24] neglected to discuss the researchers’ positioning in relation to the participants which should be considered a critical feature of completing and analysing qualitative research. Recruitment methods were deemed to be appropriate to the research question, with most studies recruiting via adverts on social media, posters and newspapers [25, 26, 28, 29] or sampling through clinician referrals [23, 24, 27] and outpatient clinics [22, 30–32]. However, most participants were those from white, middle-class backgrounds. Additionally, two studies [22, 24] presented raw data only with no details on data analysis, and a further three studies [25, 27, 29] only described this process partially.

### Data synthesis

Broadly, all interventions were considered acceptable to parents, however many were not deemed complete in their current form and had suggestions for improvement. Participants generally reported that e-Health interventions improved their mood [22, 25, 27, 29, 31], and reported benefits over traditional face-to-face therapy in terms of improved accessibility and reduced barriers. However, the lack of interpersonal communication in e-Health interventions was generally viewed negatively, with participants finding the interventions less person-centered when delivered remotely. Two overarching themes were identified across the papers; these themes included ‘Elements influencing acceptability of eHealth postnatally; mode of delivery and intervention properties’ and ‘Barriers and facilitators to using e-Health postnatally’. Each theme was made of several subthemes. The themes are displayed in Table2. The identified themes and subthemes were represented in studies across a range of differing intervention delivery modalities, with the exception of Theme 1 subtheme iv, and Theme 2 subtheme ii in which data were primarily drawn from online delivered interventions (for an overview, see Table3).

**Table 2:** Themes and subthemes arising from data synthesis

Themes	Subthemes
Elements influencing acceptability of eHealth postnatally; mode of delivery and intervention properties	i) Allowing parents control and time ii) Content and pacing preferences for parents iii) Option for coaching support during the intervention iv) A smooth and positive user experience
Barriers and facilitators to using e-Health postnatally	i) eHealth fits into parents’ routines and schedules ii) Overcoming stigma through anonymous interaction iii) Acceptability is affected by resource availability and preferences

**Table 3:** Summary of themes and subthemes present across intervention modalities

		Theme 1 Subtheme:				Theme 2 Subtheme:		
**Author**	**Modality**	**i**	**ii**	**iii**	**iv**	**i**	**ii**	**iii**
Ashford [29]	Online Self-help	X	X	X	X	X	X	
Danaher [23]	Online self-help	X	X	X		X		
Pugh [28]	Online self-help	X	X	X		X	X	
O’Mahen [26]	Online self-help	X	X	X	X	X	X	X
Avalos [27]	App self-help	X	X	X	X			
Shorey [30]	Telephone calls		X	X				X
Baker-Ericzén [22]	Telephone calls	X	X	X				X
Barrera [25]	Text message	X	X	X				X
Pugh [24]	Email			X		X		
Seshu [31]	Audio recordings	X		X		X		X
Hensel [32]	Video calls		X	X		X		X

## Theme 1: elements influencing acceptability of eHealth postnatally; mode of delivery and intervention properties

The majority of the studies discussed elements of the intervention that participants found helpful, whether this was directly related to an impact on mental health or on other areas of the participants lives.

### Subtheme i - allowing parents control and time (6 studies)

This theme relates to the concept of the remote intervention giving participants a sense of control and permission to focus on their own needs [23, 28] and the idea that the intervention was normalising [26, 28]. In relation to the parents’ mental health, participants found the strategies within the interventions helpful to improve their mood [22, 25, 27, 29, 31]. Avalos found that participants gained ‘better stress management, reduced anxiety, improved sleep and improved physical activity’ from completing the intervention [27]. Another indirectly helpful benefit of partaking in the remote intervention was an unintentional benefit to parenting where participants felt more relaxed and patient in their parenting as a result of completing the intervention, which could in part be linked to the reduced stress and removal of barriers by accessing a therapist remotely in the postnatal period [28].

### Subtheme ii - content and pacing preferences for parents (7 studies)

Participants in four studies found different elements of the therapy approach valuable, and all four of these studies used a cognitive behavioural therapy (CBT) approach [22, 23, 28, 29]. Mood tracking, behavioural activation and psychoeducation were all named by participants as the active components and useful features of the CBT interventions to improve parental mental health. Whilst the papers report using a CBT approach, it is likely that they were using specific elements of this therapy modality. Participants across the studies valued a variety of modules and having content relevant to their own circumstances [23, 26, 28, 29] and where the content or module options were limited, studies found that participants requested more unique content. For example, [26] where a participant cited, ‘I struggled to recognize things that were useful examples from my day to day life’ before discontinuing treatment. Some studies reported that participants did not find the administration of the intervention helpful, such as finding the pace of one module per week too fast [28], wanting the modules to be shorter to fit in with new parenthood [27], or wanting freedom to start the intervention at different points within the modules [27], as participants had prior knowledge in the area and finding the initial modules too basic [23, 25, 27, 28].

Interventions were delivered via a number of different remote means. Interventions that were delivered via online modules had positive responses particularly with regards to the way that tasks were broken down [23], and that the content is able to be referred to in the future, which gave participants a sense of control over their symptoms [28]. The anonymous nature of online interventions appealed to participants [26, 28], as well as the fact that they could be completed from home around participants existing, busy schedule [26, 28, 29]. Conversely, online modules without any therapist input were found to be hard to engage with due to motivation [26] and the non-personalized content, which some participants found to be irrelevant to them [26, 29], too fast and therefore challenging [28], or too slow and basic [27, 28]. Telephone and video based interventions were found to be acceptable by their users due to their similar flexibility, overcoming the barriers of physically attending appointments [22, 32], and also due to their more personalized nature, making participants feel listened to, reassured and comforted [30]. Some participants found it difficult to organize phone calls around their newborn child’s schedule [30], which is a barrier that online delivery of self-administered modules is not affected by.

### Subtheme iii - option for coaching support during the intervention (8 studies)

Seven of the eleven interventions included support from a coach or therapist, the majority of which was weekly [23, 26, 28–30, 32] and with a trained researcher [22, 23, 29], a doctoral trainee psychologist [24, 29] or a peer volunteer [30]. Across studies, participants reported positive experiences of having a therapist to speak to either as a part of, or whilst undertaking, the remote intervention [22–25, 28–30]. The coaching contact was considered useful both to encourage participation in the intervention and serve as a reminder to continue to log in and keep accountability, as well as in terms of a friendly person to support participants in a helpful and thoughtful way. Coaching support was repeatedly reported positively as reassuring and comforting, making the technological interventions more personable and therefore agreeable. However, one area of disagreement was highlighted in Shorey et al. [30] which found that the nature of the remote intervention with a peer supporter made for an awkward and superficial relationship. In studies without therapist support, this was bought up as a barrier to completing the intervention [27, 28]. Where no therapist support was offered, participants found that the impersonalized nature of the intervention added to their feelings of reduced motivation and made them want to drop out [28], and reported that personalized support to download the app and encouragement to use it was missing [27].

### Subtheme iv - A smooth and positive user experience (4 studies)

One area in which studies found the most critique from their participants was in terms of the user experience. One CBT online module intervention found that participants found the web pages not to be phone friendly which reduced participation [29], whereas other studies’ participants reported that the apps were easy to use [27]. Equally, some studies found that participants desired less free-text boxes due to finding them hard to complete and wanted drop-down boxes [29], whereas some studies participants requested more free-text boxes to make the intervention feel more personalized and ‘their own’ [26]. The intricacies of delivery of e-Health content were raised as a concern, with participants requesting improvements such as a request for the intervention audio files to be recorded by a female voice as well as a male voice [27].

## Theme 2: barriers and facilitators to using e-Health postnatally

The barriers and facilitators to using the interventions was raised by participants across all 11 articles. Across the range of articles, parent’s comments fell into three subthemes.

### Subtheme i - eHealth fits in to new parents’ routines and schedules (9 studies)

Accessibility of a remote intervention versus a face-to-face intervention was viewed positively. The theme of being able to fit a remote intervention into family obligations was also discussed [24, [26, 32] in a way that ‘traditional therapy could not have’ [28]. This is highlighted in Ashford et al., [29] where a participant noted that they could simultaneously breastfeed whilst undertaking the intervention. Participants in Danaher’s [23] study remarked on the utility of doing the intervention no matter the time of day or night, and that it was reassuring to have this flexibility in case their baby was crying and their ability to continue the intervention was impeded. O’Mahen et al., [26] found similar findings, with participants reporting that the remote nature of the intervention improved accessibility to an extent that they were able to continue with an intervention that they otherwise would have needed to discontinue due to childcare commitments. A further benefit noted in multiple studies was the time saving element of a technology intervention [31, 32], and the fact that it could be done from the comfort of one’s own home [26, 29, 32] which a participant in Hensel et al. [32] discussed made the intervention feel more ‘human’. Finally, although only raised in one study, it feels important to note that the travel cost saving element of technological interventions was raised. The importance of this fact may be impacted by the national health resource available in each study country. O’Mahen et al., [26] based in the UK found that participants valued the ‘no-cost option’ of the technological intervention compared with the costly expense of a private therapist.

Some studies reported finding that participants preferred a face-to-face intervention; O’Mahen et al., [26] reported that some participants found the flexibility of a remote intervention hard to manage, and that some participants reported preferring a ‘routine treatment schedule’. They found that this preference was particularly evident where women had difficulties maintaining motivation with the intervention. Additionally, in Seshu et al., [31] it was reported that face to face interventions are preferred in order to ‘pour your heart out’ to a therapist in a way which could not be done via technology. Despite being easier to access the intervention via technology a theme of loneliness was reported, as the postnatal period can often be a time that new parents feel isolated, and in O’Mahen et al., [26] some participants felt that technological interventions compounded this feeling. An accessibility barrier raised by Hensel et al., [32] was the technological issues that participants can face, such as video calls dropping, videos freezing and websites crashing; the issues were deemed to be normalized due to the knowledge that ‘technology isn’t perfect’, and it was reported that this was not enough of a deterrent to move away from technological interventions.

### Subtheme ii – overcoming stigma through anonymous interaction (3 studies)

Anonymity was highlighted as a key benefit of technological interventions, and studies found that participants felt reduced judgement compared with face-to-face interventions [26, 28, 29]. This was linked to participants considering the stigma of postnatal mental health difficulties, and is evidenced in [26] where some participants reported that the anonymity and privacy of an online intervention overcame the barrier of them feeling that people would ‘belittle their problems’ and think they were ‘inadequate mothers’ if they sought help face-face.

### Subtheme iii – acceptability is affected by personal preference and resource availability (6 studies)

Where future intervention development is concerned, studies found that participants often wanted one face-to-face meeting with a remote therapist/supporter before engaging with a remote intervention in order to gain some interpersonal familiarity [30, 32] and where therapeutic support was not offered, this was requested [26]. Similarly, a frequently-occurring theme for future development across studies was a request for a wider range of content which could be more tailored to individuals to choose which modules are most applicable for them [25, 26]. Barriers to participating in the interventions identified within these studies include individual’s preconception of remote interventions, including a hopeless mentality [26] and an uncertainty about the efficacy of remote treatment. For example, one participant stated; ‘I wasn’t convinced that sitting in front of a screen would work’ [32]. O’Mahen et al., [26] found that some participants preferred a face-to-face option, however in the absence of their preferred option they signed up to an online intervention, which meant they had low commitment, motivation and expectation of the intervention’s efficacy. Where resource is concerned, one study [31] based in a deprived area found that access to the technology was a theme; women were using their husbands’ mobile phones to engage with the intervention and were unable to access it whilst their husbands were at work. Similarly, participants of a telephone intervention also noted the use of their personal contracted telephone minutes which highlights that resource should be considered an additional barrier [22].

## Discussion

This systematic review is the first of its kind to consider the acceptability of e-health interventions for mental health support for parents in the postnatal period. Using broad search terms, we identified 11 qualitative studies that explored parental views on the acceptability of e-health mental health interventions during the postnatal period. Overall, the reviewed studies indicate that e-health interventions are acceptable to parents in terms of the accessibility, anonymity, usability, and helpfulness of support. However, included studies were often limited by poor reporting of intervention characteristics or diversity of population.

Despite the inclusion of perinatal e-health interventions in policy and practice, we only found 11 studies that met our inclusion criteria. There was little research among postnatal mothers, and importantly, no research addressing the perspectives of partners. This restricts the generalizability of currently work to postnatal mothers, whilst is problematic given the increasingly work around postnatal mental health needs in fathers and partners [33]. Furthermore, the majority of mother’s included within current studies were from white, middle-class backgrounds. Whilst perspectives of these mothers are important for service delivery, current research does not include the voice of the diverse population of mothers. This is particularly important, as those from minority ethnic or racial backgrounds, and from socioeconomically disadvantaged positions may not have access to the same e-health technologies [34, 35] and are at increased risk for poor mental health during the postnatal period [36, 37].

Whilst the results from this review indicate that e-health interventions for postnatal mental health appear acceptable to parents, the active components of e-health interventions have yet to be comprehensively addressed. Components of CBT were commonly described across interventions, with mood tracking, behavioural activation and psychoeducation appearing to be important aspects of e-health interventions that were considered acceptable. However, the definition and active components of these interventions require further attention. Whilst CBT may be a useful approach for the mothers included within this review, there are considerable challenges when using CBT in minority populations. For example, CBT has been developed by developed, socially dominant groups, privileging concepts such as empowerment and self-control, and overlooking cultural considerations of minority groups which may differ from the majority narrative [38]. Furthermore, whilst CBT is a useful umbrella term for the approaches used, it was challenging to pull apart the active components of CBT that were being used in the interventions. As such, this review highlights important considerations about the design of psychological interventions for postnatal mental health. Information regarding the design and the active components of e-health interventions is essential, complemented using logic models and/or psychological theory as described in the Medical Research Council framework for complex interventions [39].

Despite these limitations of the current research, there were a number of features across e-health interventions that participants found acceptable. Firstly, e-health interventions appear to benefit from an element of individualized coaching support, between coach/therapist and service user. The current review has demonstrated the impact that the addition of individualized support can have, both on the acceptability of the intervention as well as the motivation and personal responsibility to continue engaging with the treatment. Participants in the studies covered by this review also demonstrated some preference to an initial face to face meeting with their therapist support where possible, as they found this created a more personalised relationship, making the subsequent interactions feel less stilted. A researched example of this is in PTSD therapy [40]. The initial face-to-face meeting is also cited as a benefit for therapists who are able to garner information from the client that is otherwise lost via remote communication means, such as body language, and evidence of self-care [41].

E-health interventions also appear to benefit from providing individualized content, by either supplying a range of modules to choose from or allowing the user to select where in the course they begin. This was a need that was identified frequently throughout the data across many of the studies included, as some app and web module users found that the interventions were not paced well for them, or found that the content was too demanding or simplistic. These contrary opinions appeared within participants using the same apps, therefore demonstrating that individual differences in preference of therapy pace were at play, rather than an issue with the intervention itself not being designed well. Consequently, it is important to consider a range of modules and starting points for any future remote intervention design.

### Strengths & limitations of the review

The current review was conducted using a thorough, systematic searching methodology. however, the papers included were limited to those using qualitative methodology. Despite this, it was considered that studies reporting quantitative outcomes were unlikely to capture a rich understanding of parent acceptability of e-Health interventions. Additionally, the review contains a range of different methods for delivering e-Health mental health interventions, and we have attempted to draw conclusions across the spectrum. Patient concerns can differ based on different modalities of therapy delivery, and although sample sizes were too small to separate and report themes by modality, it is considered a limitation of the research. The included studies originate from a range of countries with differing guidelines on postnatal support, and therefore the benchmark of what support parents can expect to receive is also not homogenous within this review. Furthermore, included studies typically came from high income countries, therefore, generalizability to low or middle income countries is not practical, as e-Health interventions may be less accessible or more difficult to deliver across these different settings.

## Conclusion

Health systems need to adapt to meet the increasing mental health needs during the postnatal period, so there is urgent need for evidence regarding the acceptability of e-health interventions. e-Health interventions designed for mental health support in the postnatal period are well received by their users. e-Health interventions overcame a number of barriers present in face-to-face interventions including the stigma of accessing services, and competing demands of family obligations, which are particularly pertinent in the postnatal period. However, the evidence base is limited by the need to synthesize studies who have utilised a variety of different therapeutic approaches and methods of remote communication.

## Electronic supplementary material

Below is the link to the electronic supplementary material.

Supplementary Material 1

## Data Availability

All data generated or analysed during this study are included in this published article and its supplementary information files.
