# Supplementary material for: How acceptable do parents experiencing mental health challenges find e-Health interventions for mental health in the postnatal period: a systematic review
Source: BMC Pregnancy Childbirth. 2022 Oct 12;22:763. doi: 10.1186/s12884-022-05070-7 (PMC9554391; doi:10.1186/s12884-022-05070-7)
Supplement: Supplementary file 1 — Supplementary Material 1 [file 12884_2022_5070_MOESM1_ESM.docx]

**Appendix 1: Search Terms Used**

| Search terms related to ‘Postnatal’ | (postnatal OR perinatal OR postpartum OR peripartum OR antepartum OR antenatal OR prenatal OR maternal OR pregnancy OR birth OR "after birth") AND |
| --- | --- |
| Search terms related to ‘Psychological intervention’ | (Intervention OR treatment OR therap* OR self-help OR self-care OR service OR program* OR evaluation OR counseling OR counselling OR psychotherap* OR bibliotherapy OR self- treatment OR behaviour-change OR behavior-change OR CBT OR self-directed OR cognitive- behavioral OR cognitive-behavioural OR prevention OR promotion)  AND |
| Search terms related to ‘Mental health’ | (Well-being OR "mental health" OR "mental disorder" OR psychopathology OR "psychological disorder" OR anxiety OR fear OR panic OR phobia OR agoraphobia OR obsessive-compulsive OR "post-traumatic stress disorder" OR PTSD OR trauma OR stress OR depression OR affective OR mood OR emotion* OR mania OR bipolar OR unipolar OR dysthymia OR "baby blues" OR sleep OR insomnia OR psychosis OR schizophrenia OR delusional OR schizoaffective OR "eating disorder" OR anorexia OR bulimia OR binge OR psychosocial) AND |
| Search terms related to ‘Internet based’ | (Internet OR computer OR computer* OR online OR web OR e-therapy OR e-mental OR e- health OR telehealth OR telecare OR teletherapy OR telemedicine OR telemental OR technolog* OR virtual OR cyber OR cyberpsychology OR cybertherapy OR iCBT OR cCBT OR web-based OR web-guided OR web-supported OR web-delivered OR web-assisted OR web-aided OR web- facilitated OR computer-based OR computer-guided OR computer-supported OR computer- delivered OR computer-assisted OR computer-aided OR computer-facilitated OR internet-based OR internet-guided OR internet-supported OR internet-delivered OR internet-assisted OR internet-aided OR internet-facilitated OR online-based OR online-guided OR online-supported OR online-delivered OR online-assisted OR online-aided OR online-facilitated) |
| Search terms related to ‘Qualitative’ | AND  (Interview* OR qualitative) |
| Filters | Language=English  Date= No limiter |

**Appendix 2. Quality Assessment of Included Studies Using the Critical Appraisal Skills Programme Tool**

| Paper Title & Author | Was there a clear statement of the aims of the research? | Is a qualitative methodology appropriate | Was the research design appropriate to address the aims of the research? | Was the recruitment strategy appropriate to the aims of the research? | Was the data collected in a way that addressed the research issue? | Has the relationship between researcher and participants been adequately considered? | Have ethical issues been taken into consideration? | Was the data analaysis sufficiently rigorous? | Is there a clear statement of findings? | How valuable is the research? |
| --- | --- | --- | --- | --- | --- | --- | --- | --- | --- | --- |
| Ashford (2018) [1] | Y | Y | Y | Y | Y | Can’t tell | Y | Can’t tell | Y | Y |
| Baker-Ericzén, (2012) [2] | Y | Y. | N | Y | Can’t tell | Can’t tell | Y | N | Y | Y |
| Barrera (2010) [3] | Y | Y | Y | Y | Y | Can’t tell | Y | Can’t tell | Y | Y |
| Danaher (2013) [4] | Y | Y | Y | Y | Y | Can’t tell | Y | Y | Y | Y |
| Pugh (2014) [5] | Y | Y | Y | Y | Y | Y | Y | N | Y | Y |
| Pugh (2015) [6] | Y | Y | Y | Y | Y | Can’t tell | Y | Y | Y | Y |
| Shorey (2019) [7] | Y | Y | Y | Y | Y | Can’t tell | Y | Y | Y | Y |
| O'Mahen (2015) [8] | Y | Y | Y | Y | Y | Can’t tell | Y | Y | Y | Y |
| Seshu (2020) [9] | Y | Y | Y | Y | Y | Somewhat | Y | Y | Y | Y |
| Avalos (2020) [10] | Y | Y | Y | Y | Y | Can’t tell | Y | Can’t tell | Y | Y |
| Hensel (2020) [11] | Y | Y | Y | Y | Y | Can’t tell | Y | Y | Y | Y |
